# Confocal scanning of intervertebral disc cells in 3D: Inside alginate beads and in native microenvironment

**DOI:** 10.1002/jsp2.1106

**Published:** 2020-07-15

**Authors:** Paula A. Hernandez, Timothy D. Jacobsen, Zahra Barati, Nadeen O. Chahine

**Affiliations:** ^1^ Department of Orthopaedic Surgery University of Texas Southwestern Medical Center Dallas Texas USA; ^2^ Department of Biomedical Engineering Columbia University New York New York USA; ^3^ Department of Orthopedic Surgery Columbia University New York New York USA

**Keywords:** 3D, alginate beads, cell morphology, confocal imaging, cytoskeleton, nucleus pulposus

## Abstract

The interaction between cells and their extracellular matrix (ECM) is crucial to maintain both tissue and cellular homeostasis. Indeed, cell phenotype is significantly affected by the 3D microenvironment. Although highly convenient, isolating cells from the intervertebral disc (IVD) and growing them in 2D on plastic or glass substrates, causes them to rapidly lose their phenotype and consequently alter their gene and protein expression. While characterization of cells in their native or simulated 3D environment is preferred, such approaches are complexed by limitations in phenotypic readouts. In the current article, we describe a detailed protocol to study nucleus pulposus cells in 3D—embedded in alginate as a permeable cell‐staining reservoir, as well as adaptation for cell staining and imaging in their native ECM. This method allows for detection of phenotypical and cytoskeletal changes in cells within native tissue or 3D alginate beads using confocal microscopy, without the need for histological processing.

## Graphical abstract

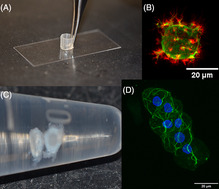

In this article, we explain easy to adopt protocols that permit visualization of cells in their native matrix or biomimetic 3D system, including (A) how to build an inexpensive and easy to assemble sample holder that allows confocal visualization of cells embedded in alginate beads (B), and how to process rodent nucleus pulposus (C) for better staining and confocal visualization of cells in situ (D). Both without the need of further histological processing.

## 1 INTRODUCTION

Sparsely populated tissues composed of large amounts of extracellular matrix (ECM), such as the nucleus pulposus (NP) and articular cartilage, are challenging when isolating cells for experimentation. The limitations associated with low cell yield can be overcome by expanding the cells in a 2D culture. During this process, cells experience a phenotypic instability, where the lack of an ECM‐rich three‐dimensional (3D) microenvironment, together with extended passaging, changes the synthesis of collagen type II to collagen type I, resulting in a shift toward a fibroblast‐like phenotype.^1^

The use of 3D models in cell culture is becoming increasingly popular due to the growing understanding of the crucial interaction between the cell and its immediate niche,^2^ the mechanical forces influencing the phenotypical outcome,^3^ and the effect of substrate elasticity on signaling pathways, phenotypic expression and cytoskeletal response.^2^, ^4^, ^5^, ^6^, ^7^

Alginate, a hydrogel obtained from brown algae, has been largely used as a non‐toxic, inexpensive and easy to use 3D hydrogel model. Beads of alginate‐embedded cells are formed when the cell‐alginate mixture contacts a calcium solution and can be kept stable for several weeks in culture. Polymerized 1.2% alginate hydrogel is soft, with a stiffness of about 5 to 10 kPa.^8^ Even though cells do not attach to the alginate, cells form attachment with newly deposited ECM while embedded in alginate. In fact, several reports have shown that cells regain their normal phenotype and revert synthesizing type II collagen, the physiologically relevant type of collagen, after days of culture in alginate hydrogels.^1^, ^9^, ^10^, ^11^, ^12^, ^13^, ^14^, ^15^, ^16^, ^17^, ^18^ Other hydrogel systems, such as agarose, have also been used and shown effective for cell differentiation.^19^, ^20^, ^21^ However, unlike agarose hydrogels, cells can be released from alginate hydrogels after culture and used for further analysis. For this reason, we have developed an approach to use alginate beads as a permeable cell‐staining reservoir, where cells can be encapsulated, stained and imaged while inside the bead using similar steps to those typically used in immunofluorescence of cells in 2D.

Being able to observe cell morphology and phenotype directly in their 3D environment is key for detecting subtle changes. Numerous studies have attempted to visualize cells in alginate beads by a variety of histological processing, such as paraffin embedding,^22^, ^23^, ^24^, ^25^, ^26^ cryostat sectioning,^27^, ^28^, ^29^ polyethylene‐glycol (PEG) embedding,^30^, ^31^ LR white resin embedding^32^ releasing cells from beads before immunostaining,^33^ or processed for electron microscopy.^1^, ^10^, ^34^ Some studies have performed direct fluorescent microscopy in beads stained with calcein using low magnification,^35^ while others have performed two‐photon laser scanning microscopy,^36^, ^37^ but even this method yields insufficient image resolution for observation of subtle changes in cell phenotype and morphology.

While glass bottom plates are useful for imaging flat surface samples (eg, thin layer of tissue), a round bead is difficult to keep contained in place in a flat bottom sample holder, which challenges image acquisition of hydrogel beads. To overcome this problem, we describe an inexpensive sample holder—that can be made with materials readily available in wet laboratory settings—to facilitate the imaging of alginate beads using confocal microscopy.

When studying cell morphology and ECM in tissue, the conventional approach has been to process the tissue samples for histological sectioning and staining, either by immunohistochemistry or by fluorescence immunostaining. While these approaches can indeed provide extensive information on matrix arrangement and abundance in less dense opaque tissues, such as cartilage,^38^ they can be time and resource consuming. Nucleus pulposus is a much softer tissue with better light transmittance, which gives great potential for a rapid characterization of cell phenotype in situ. The protocol described herein provides an alternative approach, which can be used to compliment or in lieu of morphological analysis using standard histological approaches.

For demonstration purposes, we have used F‐actin staining with phalloidin as a tool to visualize cell phenotype and actin cytoskeletal arrangement in both beads and tissue. Depending on whether the aim is to study NP cells in 3D models or in their native matrix, these protocols for sample preparation for confocal scanning have the benefit of permitting a larger sample depth. This allows us to better understand cells behavior and distribution in a larger tissue area of a 3D model compared to the 5 to 10 μm thickness typically obtained in histological slides.

## 2 MATERIALS

### 2.1 Cell source

Nucleus pulposus (NP) cells can be isolated from the intervertebral discs (IVD) of a variety species (eg, bovine, porcine, rodent, etc.), and there are many different isolation protocols in literature.^14^, ^39^ While recommending an optimal protocol is beyond the scope of this study, we describe here the protocol used for isolating bovine NP cells. Briefly, lumbar spines from freshly slaughtered juvenile cows were obtained from an abattoir (Green Village Packing Company, Green Village, NJ; permission was obtained to use these animal parts for research). Bovine NP tissue was dissected from multiple lumbar spinal levels, and then pooled, minced and digested in complete media (high glucose DMEM + 10% FBS + 1% antibiotic‐antimycotic solution) supplemented with 0.3 mg/mL collagenase type I (Sigma‐Aldrich) and 0.3 mg/mL collagenase type II (Sigma‐Aldrich) for 3 hours at 37°C with gentle agitation. Cell digest was passed through a 70 μm cell strainer, washed with PBS, counted and grown in complete medium as described below.

For imaging of cells in situ in rodent discs, mice and rat sentinel animals, donated from the Animal Research Center at UT Southwestern, were used. Tissue was dissected and used within 4 hours of euthanasia. We used one young male mouse (14.36 g, C57BL, ~4 weeks old), one adult male mouse (27.20 g, C57BL, ~13 weeks old) and one adult male rat (382 g, Sprague Dawley, unknown age).

### 2.2 For tissue culture

Complete media consisted of DMEM high glucose with sodium pyruvate and glutamine (Sigma, D6429), 10% fetal bovine serum (heat‐inactivated preferred; Sigma, F4135), 50 μg/mL L‐ascorbic acid 2‐phosphate trisodium salt (Wako, 323‐44822), 1% antibiotics and antimycotics solution (Gibco, 15240062).

### 2.3 For alginate solution and preparation of cell‐embedded beads

Alginic acid sodium salt from brown algae, medium viscosity (Sigma, A2033); 150 mM NaCl + 10 mM HEPES (adjust to pH 7.4 and autoclave); 102 mM CaCl_2_ + 145 mM NaCl + 10 mM HEPES pH 7.4 (autoclave); 50 mM sodium citrate + 150 mM NaCl + 10 mM HEPES (adjust to pH 7.4 and autoclave). 100 mm Petri dishes, 21‐Gauge needles, syringe filters (Millipore SLGP033RS, low binding, pore size 0.22 μm), and 1‐ or 3‐mL syringes.

### 2.4 For fluorescent staining

Phosphate Buffer Saline (PBS, Sigma), Hanks' balanced salt solution (HBSS no phenol red, Gibco) supplemented with 1.26 mM CaCl_2_ and 400 μM MgSO_4_ (we call this solution HBSSCM); 4% paraformaldehyde (PFA) prepared in PBS, 4% paraformaldehyde prepared in HBSSCM, Tween‐20, Triton‐X100, Bovine Serum Albumin (BSA), Goat's serum (Gibco), Phalloidin‐iFluor 555 (Abcam, ab176756), Phalloidin‐ Alexa Fluor 488 (Invitrogen, A12379), primary and secondary antibodies, DAPI (Invitrogen, D1306).

### 2.5 For building the bead and small tissue sample holder

22 mm × 50 mm No. 1.5 coverslips, 200 μL pipette tips, microtome blade, White Gorilla glue pen for glass (superglue).

### 2.6 To image larger tissue in confocal microscope

35‐mm with 12‐mm round glass bottom Petri dish and a coverslip.

## 3 METHODS

### 3.1 Protocol 1: Cell culture in alginate beads

1. Isolate nucleus pulposus cells from the tissue source of interest.
2. If needed, cells can be expanded by growing them in a tissue culture flask or dish with complete media for a few days. Multiple passaging is not recommended, as cells start losing their phenotype shortly in culture. Freshly isolated cells or up to passage 2 yield best results.
3. Prepare 1.2% alginate following a previously described protocol with some modification.^11^ For this, dissolve alginate powder in 150 mM NaCl + 10 mM HEPES pH 7.4. This solution dissolves faster at 37°C with constant agitation.
4. Sterilize by filtration, as autoclaving may affect stiffness of the polymerized beads. Use a 0.22 μm syringe filter and a 1‐ or 3‐mL syringe. We have observed that the larger the syringe, the more difficult it is to filter the alginate solution. Store sterile solution at 4°C for up to 2 weeks. Pre‐warm the alginate solution to 37°C before use for cell embedding.
5. Release cells from culture dish using trypsin or other trypsin‐free method. Freshly isolated cells from tissue can be used as well. Count cells using hemocytometer or equivalent. Add cells to alginate mixture to arrive at a final density of 2 × 10^6^ cells/mL (see NOTE 1). To do this, centrifuge cells after counting and calculate volume of alginate needed to resuspend them to achieve the desired cell density. Using a 1000 μL micropipette, carefully and slowly resuspend the cell pellet in alginate solution. To avoid bubbles, do not pipette the whole volume up and down.
6. Once the cell suspension is homogenous in the alginate solution, place 20 mL of polymerizing (CaCl_2_) solution in a 100‐mm petri dish. Depending on the final volume, load alginate‐cell suspension into a 1‐ or 3‐mL syringe with a 21G needle.
7. Slowly take the mixture of cells in alginate and dispense dropwise into the polymerizing solution while gently shaking the petri dish by hand. Each drop will form a bead immediately after touching the calcium.
8. After dispensing all the volume in several drops, leave the beads to further polymerize in the incubator at 37°C for an additional 10 minutes.
9. Wash the beads twice for 2 minutes each with gentle shaking using 150 mM NaCl + 10 mM HEPES pH 7.4. Tilt the petri dish and aspirate from the top and sides, avoiding beads.
10. Give a final wash with complete media and then add complete media to start cell culture (see NOTE 2).

### 3.2 Protocol 2: Building sample holder to visualize alginate beads directly in the confocal microscope

**FIGURE 1:**
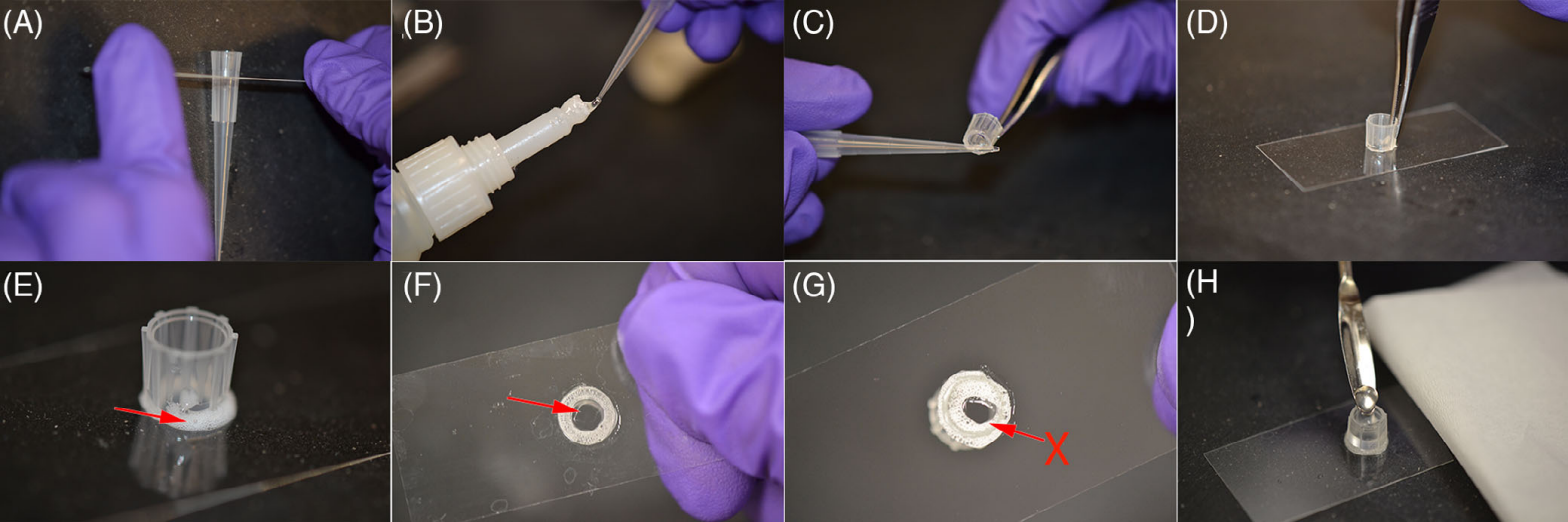
Procedure to build the device to visualize beads in confocal microscope. A, Cut the tip at 5 to 6 mm from the top, B, C, using another tip, apply a small amount of superglue for glass into the cut section of the tip (D) place the cut tip at the center of a coverslip, apply some pressure with the back of the forceps for a few seconds, E, wait for the superglue to cure, small bubbles will form. F, Make sure to leave a central round area large enough to allow confocal imaging. G, If too much glue is applied, the glue will expand blocking the view. H, When ready to image cells with confocal microscope, add a small amount of HBSSCM to prevent dehydration and then a single bead, a small section of tissue or a rodent IVD

1. Clean the glass coverslip with 70% alcohol.
2. Cut a 200 μL pipette tip at about 5 or 6 mm from the top (see Figure 1A). We have observed that a microtome blade is the most effective at making a clean and straight cut.
3. Use the tip of a 200 μL micropipette tip to add a small amount of superglue into the cut part of the tip (Figure 1B,C).
4. Using forceps, carefully place the tip with the superglued side facing the coverslip, at the center of the coverslip (Figure 1D).
5. Apply a small pressure using the back section of the forceps. Avoid moving the tip along the glass surface.
6. Leave the sample holder to dry for at least 30 minutes at room temperature. Small bubbles will form (Figure 1E).
7. Make sure to leave enough area free of glue at the center of the sample holder. The use of too much glue will interfere with the area at the center of the cylindrical tip needed for placing and imaging specimens (Figure 1F,G).
8. Once dried, place a single alginate bead inside the sample holder using a small spatula (Figure 1H).
9. Add a small volume of HBSSCM to prevent dehydration of the bead.
10. The bead in the holder is ready to be transferred to a microscope stage for imaging.
11. Follow standard microscope protocol for imaging.
12. To remove the bead from the holder at end of imaging, use the same small spatula to transfer the bead.
13. Rinse the holder with water and let air dry. The holder can be reused several times.

### 3.3 Protocol 3: Immunostaining of alginate beads

**FIGURE 2:**
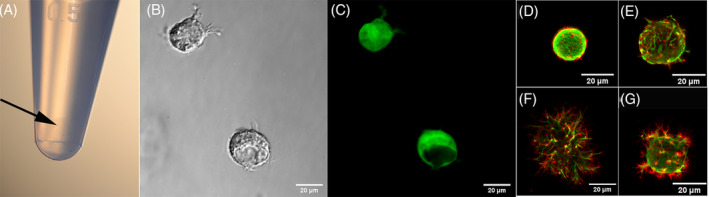
Representative images of cell staining and imaging in alginate beads. A, Beads can be seen at the bottom of the tube with an external light. Care must be taken not to pipette the beads. B, Differential Interference Contrast (DIC) image of two nucleus pulposus cells inside a bead. C, Confocal image of Calcein AM (green) staining of the same cells in B. D‐G, Confocal images of cells in beads stained for F‐Actin (Phalloidin, red) and vimentin (green) of untreated cells (D) and cells after inflammatory treatment with TNF‐α (E‐G), maximum intensity projection of z‐stack. Sections are 1 μm apart. Scale bars are 20 μm. Images were taken with a ×40 objective

1. For immunofluorescence analysis of cells cultured in beads, all solutions were prepared in Hanks' Balanced Salt Solution (HBSS) supplemented with 1.26 mM CaCl_2_ and 400 μM MgSO_4_ (HBSSCM). The use of PBS with no calcium or magnesium will result in dissolution of the alginate beads (see NOTE 3).
2. To prepare 4% PFA in HBSSCM: weigh PFA using adequate eye, skin and respiratory protection. Pre‐warm a solution of HBSSCM—this can be done in a microwave, avoid boiling. Transfer to a chemical hood and add the PFA powder while stirring the solution with a magnetic stirrer. Cover with parafilm or aluminium foil and continue stirring until the powder is completely dissolved. Filter 4% PFA‐HBSSCM with a 0.22 μm syringe filter, make 1 to 2 mL aliquots and freeze in −20°C.
3. Before use, warm the PFA‐HBSSCM aliquot to 37°C.
4. Carefully transfer alginate beads using a small spatula to a 1.5 mL microcentrifuge tube (Figure 2A) with 4% PFA‐HBSSCM at a ratio of 200 μL per bead.
5. Fix at room temperature for 15 minutes with gentle shaking to help diffusion.
6. After fixation, wash beads two times for 5 minutes each in HBSSCM.
7. If needed, permeabilize cells with 200 μL per bead of 0.1% Triton X‐100 prepared in HBSSCM for 5 minutes at room temperature while gently shaking.
8. Wash two times for 5 minutes each in HBSSCM in 500 μL per bead.
9. Prepare the blocking solution (5% BSA in HBSSCM + 10% goat serum; syringe filter using 0.22 μm filter). Incubate beads in 200 μL per bead of blocking solution at room temperature for 1 hour.
10. Prepare primary antibody in buffer containing HBSSCM + 5% BSA (syringe filtered) and incubate overnight at 4°C in a 1.5 mL tube, using the manufacturer's recommended or otherwise optimized dilution factor. We used anti‐vimentin (1:1000, Abcam ab8069) (see NOTE 4).
11. Wash the beads three times for 10 minutes each with HBSSCM + 0.2% Tween‐20. Use 500 μL per bead and gentle rocking (see NOTE 5).
12. Add secondary antibody prepared in buffer (same as used in step 10), for 1 hour at room temperature in the dark, using the manufacturer's recommended or otherwise optimized dilution factor. We used anti‐mouse Alexa Fluor 488 (1:500, Invitrogen A‐11001) as secondary antibody or Phalloidin‐iFluor 555 to stain F‐actin (1:1000 Abcam ab176756).
13. In a dark space, wash beads three times for 10 minutes each with HBSSCM + 0.2% Tween‐20. Use 500 μL per bead and gentle rocking (same as in step 11).
14. If needed, nuclei can be counterstained using DAPI or any other equivalent staining. For DAPI, prepare a fresh dilution of 300 nM in HBSSCM and incubate the beads for 5 minutes at room temperature using 100 μL per bead.
15. Wash beads once for 5 minutes with HBSSCM (500 μL per bead).
16. To visualize cells in confocal microscope, carefully transfer one bead to the sample holder (see Protocol 2).
17. Alternatively, cells can be released from beads and analyzed using imaging flow cytometry (eg, Amnis Imagestream) or imaged using other methods. To dissolve the beads, add 55 mM sodium citrate + 150 mM NaCl + 10 mM HEPES pH 7.4 for 10 to 15 minutes and gently mix. Centrifuge the released cells at 500 g for 10 minutes and resuspend cell pellet in a small volume of PBS (see NOTE 6). Cells may be plated on poly‐L‐lysine coated coverslips. Leave for about 30 minutes at room temperature and then mount with mounting media, following standard protocol.
18. Image cells using fluorescence imaging of choice (eg, confocal microscopy). We imaged bovine cells with laser scanning confocal microscope Olympus XL70 using a ×40 objective as a Z‐stack with 1 μm space between slices (Figure 2B‐G).

### 3.4 Protocol 4: Visualizing cells in thin slices of bovine nucleus pulposus

**FIGURE 3:**
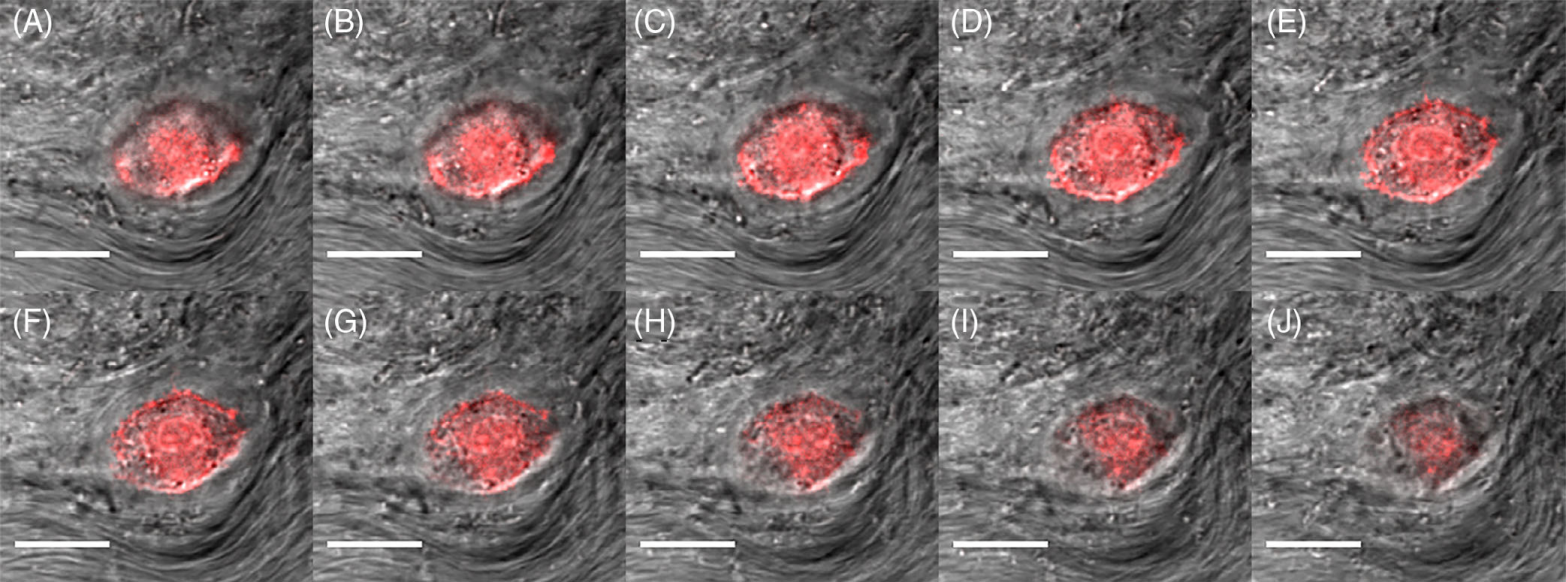
Representative Z‐stack of a bovine nucleus pulposus cell in native tissue. A‐J, Bovine tissue was stained for F‐Actin (Phalloidin, red) and visualized with confocal microscope. DIC (gray) are overlaid. Sections are 1 μm apart. The native matrix can be appreciated in DIC images. Scale bars are 20 μm. Images were taken with a ×40 objective

1. Using a biopsy punch or scalpel blade, isolate a small section of bovine nucleus pulposus tissue.
2. Using a microtome blade and forceps carefully cut a very thin section of tissue. Given the consistency of bovine nucleus pulposus, we typically generate sections of about 1 mm thick × 5‐6 mm wide. Given the dense ECM of the tissue that can reduce penetration of the dye, we do not recommend using sections thicker than 1.5 mm.
3. Transfer the tissue to a 1.5 mL microcentrifuge tube and fix with 1 mL of 4% PFA in PBS for 15 minutes at room temperature and gentle rocking to facilitate diffusion.
4. Wash the slice of tissue twice for 10 minutes each with 1 mL of PBS and gentle shake.
5. If needed, permeabilize the tissue with 0.5% Triton X‐100 in 1 mL of PBS for 10 minutes at room temperature.
6. Repeat wash step 4.
7. Add blocking solution (5% BSA, 10% goat serum, PBS, syringe filtered) for 1 hour at room temperature and gentle rocking.
8. Add 500 μL of phalloidin conjugated with iFluor 555 (or alternate stain of choice) at a dilution of 1:100 in 5% BSA prepared in PBS (filtered) overnight at 4°C with gentle shaking (see NOTE 7)
9. Wash the tissue three times with 1 mL of PBS for 10 minutes each.
10. Add 500 μL of 300 nM DAPI if necessary. Incubate for 5 minutes at room temperature. Wash once with 1 mL of PBS for 5 minutes.
11. Place the slice of tissue in the center of a 35 mm bottom glass Petri dish.
12. Add a small volume of PBS to prevent dehydration of tissue.
13. Cover with a small round or square coverslip and apply a slight pressure. Placing the coverslip on top will prevent the sample from moving while imaging.
14. The tissue is ready to be imaged by confocal microscopy (Figure 3A‐J).
15. If tissue is small enough, the same device used in Protocol 2 can be used (see Protocol 5).

### 3.5 Protocol 5: Visualizing cells in mouse and rat IVD

**FIGURE 4:**
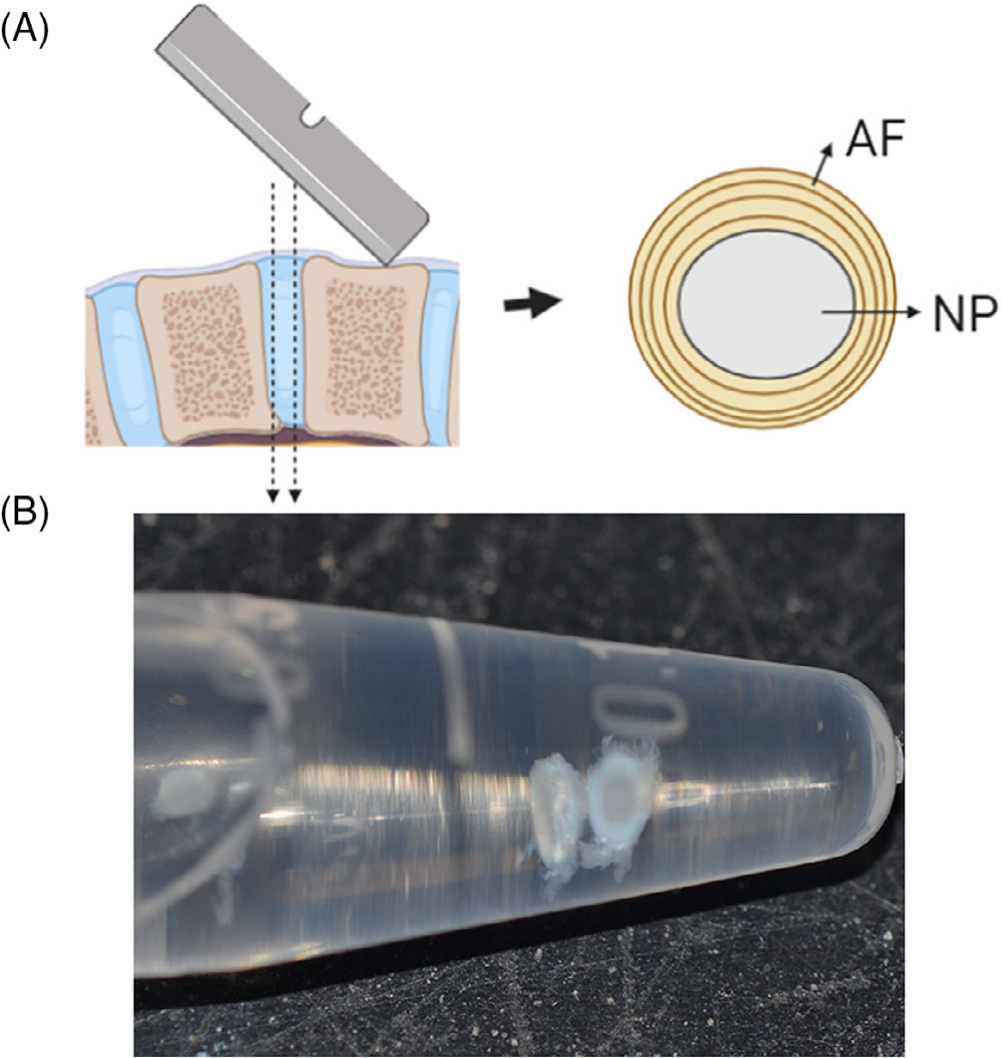
Diagram for extraction of annulus fibrosus (AF) with nucleus pulposus (NP) sections from mice and rat. A, With a microtome, cut at both ends of a disc, avoiding the end plates. Tissue should be 2 mm maximum. Leave NP surrounded by AF for better handling. B, Image of resulting discs fixed in 4% PFA

**FIGURE 5:**
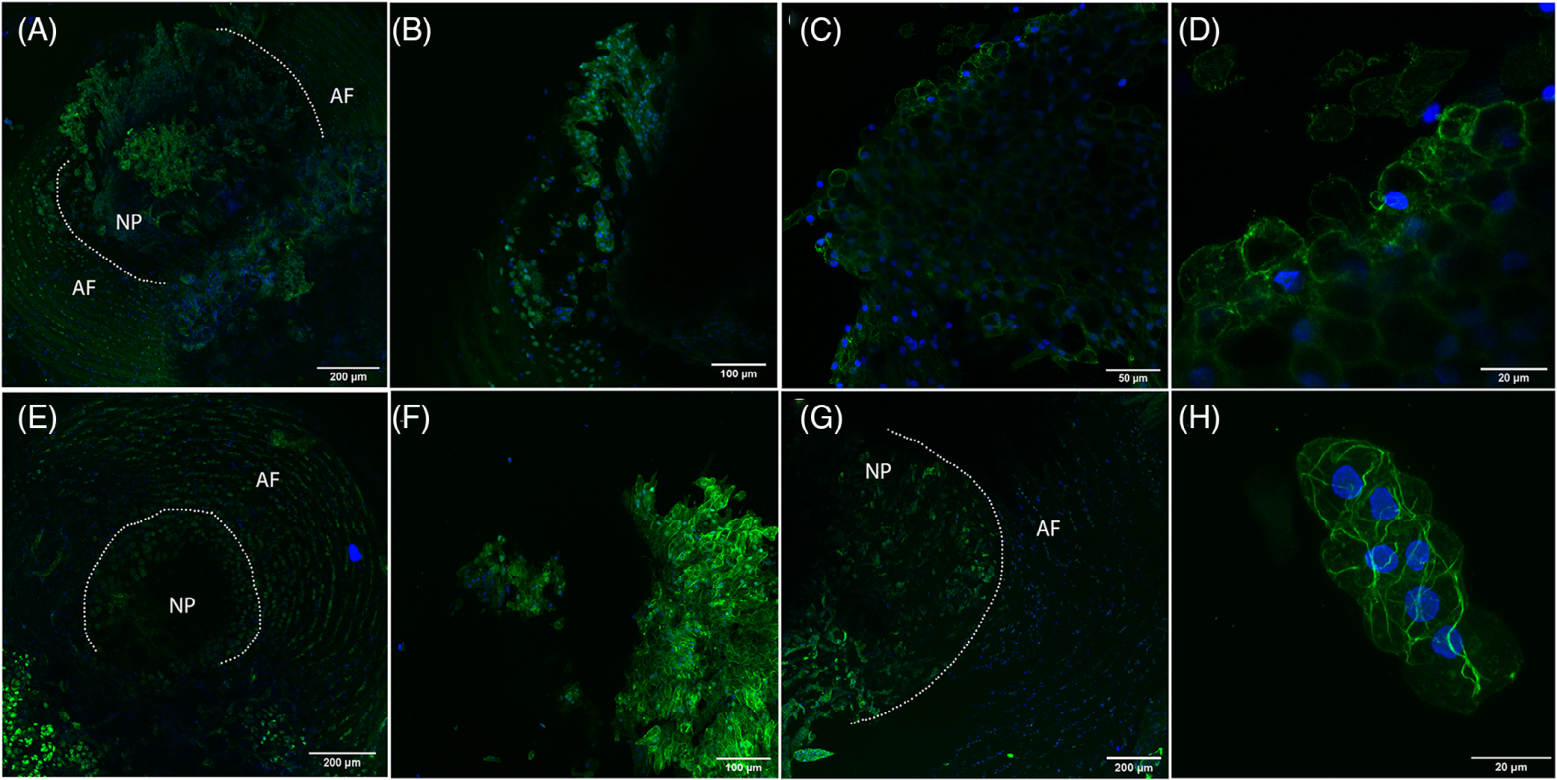
Representative images of mice and rat IVDs. Tissues were fixed and stained with Phalloidin‐Alexa Fluor 488 for F‐Actin in green and DAPI for nuclei in blue. A, Maximum intensity projection of a young mouse NP and AF imaged with a ×10 objective, sections are 10 μm apart. B, Maximum intensity projection of a young mouse NP imaged with a ×20 objective, sections are 2 μm apart. C, Single image of a young mouse NP obtained with a ×40 objective. D, Zoom from image in (C). E, Maximum intensity projection of an adult mouse NP and AF imaged with a ×10 objective, sections are 5 μm apart. F, Maximum intensity projection of an adult mouse NP imaged with a ×20 objective, sections are 2 μm apart. G, Maximum intensity projection of an adult rat NP and AF imaged with a ×10 objective, sections are 10 μm apart. H, Maximum intensity projection of an adult rat NP imaged with a ×40 objective, sections are 2 μm apart. Scale bars are 200 μm for A, E, and G; 100 μm for B and F; 50 μm for C and 20 μm for D and H

1. Isolate IVD area consisting of annulus fibrosus (AF) and nucleus pulposus from mouse or rat tail (see NOTE 8). We recommend using a dry microtome blade to obtain a clean cut below the rostral and above caudal end plates, to obtain a ring of AF surrounding NP with no end plates (Figure 4). Given the dense ECM of the tissue, we do not recommend using sections thicker than 1.5 mm (see NOTES 9 and 10).
2. To prevent tissue dehydration when isolating several IVDs at the same time, IVDs can be placed in hypertonic media (DMEM supplemented with 110 mM NaCl) until they can be fixed in 4% PFA. The hypertonic media will prevent tissue swelling and damage to the nucleus pulposus.
3. Fix tissues with 500 μL of 4% PFA in PBS per IVD for 15 minutes at room temperature with gentle rocking.
4. Wash the tissue twice with 1 mL PBS for 10 minutes each.
5. If needed, permeabilize the tissue with 500 μL of 0.5% Triton X‐100 in PBS per IVD, for 5 minutes.
6. Wash tissue twice with 1 mL PBS for 10 minutes each.
7. Add blocking solution (5% BSA, 10% goat serum, PBS, syringe filtered) for 1 hour at room temperature with gentle rocking.
8. Add 100 μL of phalloidin conjugated with Alexa Fluor 488 (or alternate stain of choice) per tissue section at a dilution of 1:100 in 5% BSA prepared in PBS (filtered) overnight at 4°C and gently shake.
9. Wash the tissue three times with 1 mL of PBS for 10 minutes each.
10. Add 500 μL of 300 nM DAPI per section for nuclei counterstain if necessary. Incubate for 5 minutes at room temperature.
11. Wash once with 1 mL of PBS for 5 minutes.
12. Carefully place the tissue at the bottom of the sample holder used in Protocol 2. Add a small volume of PBS to prevent dehydration and make sure the sample stays at the bottom.
13. The sample is ready to be imaged in a confocal microscope. For this, we used a Zeiss LSM880 Airyscan with ×10, ×20, and ×40 objectives (Figure 5).

### 3.6 NOTES

1. We have observed that 2 × 10^6^ cells/mL yield a cell density good enough to image single cells in higher magnification and multiple cells per field of view in lower magnification.
2. Cells can be maintained alive for several weeks in this system. We have observed that the addition of 10% Fetal Bovine Serum (FBS) or 10 ng/mL of TGFβ3 promote cell viability, proliferation and ECM synthesis.
3. If cells need to be tested for viability, you may add 4 μM calcein in HBSSCM for 20 minutes. Then wash beads twice with HBSSCM.
4. To minimize the waste of antibodies, beads can be incubated in 100 μL final volume. We have observed that that beads can be stained simultaneously using 100 μL resulting in consistent staining and good imaging.
5. Using an external light source facilitates the washes as beads become more visible.
6. If cells in beads are needed for other outcomes, such as RNA extraction, wash the beads for 10 minutes in HBSSCM, then add 10 volumes of 55 mM sodium citrate solution following step 17 in Protocol 3. After dissolving beads, centrifuge cells for 5 minutes at 1000 g. Wash cells with PBS, centrifuge and repeat wash with PBS. Resuspend the cell pellet in lysis buffer (we use Qiazol from QIAGEN) and proceed with RNA extraction protocol following manufacturer's instructions.
7. Given the lower permeability of tissue, we prepared phalloidin solution 10 times more concentrated than for staining beads.
8. Bovine NP is more fibrous than the well‐hydrated and gelatinous rodent NP.^40^ This makes handling of bovine tissue easier. When using mouse or rat tissue, keeping the AF surrounding the NP facilitates handling.
9. Do not place the IVD in isotonic liquid (media or PBS), as the nucleus pulposus tissue sample will swell.
10. We have utilized the four most rostral IVDs in the tail (caudal discs), as they have a larger nucleus pulposus that facilitate tissue isolation and handling.

## 4 DISCUSSION

The main advantage of visualizing cells directly in alginate beads and tissue is the ability to identify subtle cell phenotypical changes in detail, in an undisturbed microenvironment, keeping the expected cell shape and cytoskeletal arrangement of cells growing in a 3D construct. We have observed that under certain pro‐inflammatory conditions, NP cells extend a number of cell processes, which can be severed or destroyed if cells are either released from beads for staining before fixing.

Alginate beads promote a round cell morphology. Therefore, this approach is preferable for naturally round cells such as NP or chondrocytes. However, this cell embedding approach does not facilitate imaging of cells that are naturally elongated, such as AF cells.

The sample holder described here successfully holds a single bead and secures it in place, which facilitates image acquisition in a confocal microscope. A z‐stack of good resolution images can be obtained providing a detailed view of the 3D actin cytoskeleton and morphological features.

If the purpose is to visualize cell interaction with ECM in alginate beads, cells embedded within alginate can be cultured for longer durations to promote ECM synthesis. This can be achieved in a matter of days to weeks.^15^, ^18^

The reagents used in this method are the same used in standard immunofluorescence in 2D. In a 12‐mm diameter coverslip we regularly use about 80 μL of antibody solution, which can be scaled up to 120 μL for a 22‐mm round coverslip. Here, we used similar volumes and incubation times, making this method easy to adapt while maintaining a more physiological cell microenvironment. Beads and tissue can be stained and washed in 1.5 mL centrifuge tubes, scanned in confocal microscope and recovered afterwards if additional staining is needed.

The DIC images that we were able to obtain in fixed tissue, without standard histological processing, show a detailed arrangement of the ECM that surrounds the cell. Commonly used protocols for paraffin embedding or other processing steps used to generate histology sections can be harsh on delicate matrix structures and affect antibody affinity requiring extra steps for antigen retrieval. Even though we are not proposing to replace the regular histological processing and staining in cases where this procedure is scientifically warranted, our protocol for NP explants allows for cell morphology to be quickly detected in small sections of tissue. This could potentially be used to rapidly analyze cell morphology in biopsies of NP tissue originating from patients, to screen for morphological changes and decide if further histological processing is warranted. This can be especially helpful in settings where time and resources are limited.

Both bead and tissue imaging protocols described here also allow for image acquisition deeper within the sample, resulting in more than 100 μm‐depth area of analysis with a ×40 objective, and additional information on cell‐ECM space. We have not yet tested antibody detection on thin sections of NP explants, however, we expect that antibodies for highly abundant cellular proteins can be easily employed. Optimal concentration of antibody needs to be determined empirically in a case‐by‐case basis.

## 5 CONCLUSIONS

These easy to adopt protocols permit visualization of cells in their native matrix or biomimetic 3D system (alginate beads). The cell morphological changes identified using the alginate bead protocols described herein are closer to those occurring in situ than when cells are imaged in 2D. Images of cells surrounded by native ECM are useful to understand structural characteristics of the matrix and cell‐ECM interactions.

## CONFLICT OF INTEREST

All authors declare no conflicts of interest.

## AUTHOR CONTRIBUTIONS

All authors helped draft this manuscript and approved the final version. PAH designed the sample holder to visualize beads and tissue in confocal microscope.
